# *Rickettsia aeschlimannii*: A New Pathogenic Spotted Fever Group Rickettsia, South Africa

**DOI:** 10.3201/eid0808.020199

**Published:** 2002-08

**Authors:** Anne-Marie Pretorius, Richard J. Birtles

**Affiliations:** * University of the Free State, Bloemfontein, South Africa and †University of Liverpool, Liverpool, England

**To the Editor:** Spotted fever group rickettsiae are increasingly recognized as agents of disease in residents of and tourists to South Africa (1). To date, two species, *Rickettsia conorii* and *R. africae*, which cause Mediterranean spotted fever (MSF) and African tick-bite fever (ATBF), respectively, have been associated with human disease in the region; ATBF is more frequently associated with travel (1). As different antibiotic regimens are recommended for the two syndromes, differentiating MSF from ATBF is important. Increasing evidence shows that the syndromes can usually be differentiated through clinical manifestations and epidemiologic characteristics (1).

We recently encountered a South African patient who, on returning from a hunting and fishing trip, discovered a *Rhipicephalus appendiculatus* tick attached to his right thigh and an eschar around the attachment site. The patient was aware of the risk of tick-transmitted disease; after removing the tick, immediately self-prescribed doxycycline. No further symptoms developed. However, as a precaution, the patient went to a local clinic, where a skin biopsy was taken from the eschar. This sample, together with the removed tick, was submitted to our laboratory. DNA extracts, prepared from an eschar biopsy and the tick, were incorporated into a polymerase chain reaction (PCR) assay specifically targeting a fragment of the rickettsial *omp*A (2). Sequence analysis of the amplification products showed both to be identical and to share >99% similarity with the *omp*A of *R. aeschlimannii*, a species not previously associated with human disease. Unfortunately, blood samples could not be collected at the time the patients first had symptoms; thus, investigation of a disseminated infection by PCR and serologic testing was not possible.

Although genotypically indistinguishable organisms had previously been detected in *Hyalomma marginatum* collected in Portugal and Zimbabwe, *R. aeschlimannii* was first characterized following its isolation from *H. marginatum* ticks in Morocco (3) and recently in Niger (4). This encounter was the first demonstration of its presence in South Africa and in *Rhipicephalus* ticks.

A lack of suitable clinical material prevented full evaluation of the pathogenic potential of *R. aeschlimannii* in this patient and prompt antibiotic intervention may have prevented evolution of the syndrome. Nonetheless, that *R. aeschlimannii* was transmitted to the patient and established a local infection leading to eschar formation provides clear, albeit preliminary, evidence of its virulence. Until further cases are encountered, allowing better characterization of the clinical manifestations associated with *R. aeschlimannii* infection and considering the agent capable of inducing either MSF or ATBF-like manifestations is crucial; neither of these syndromes can be associated with a specific causative agent without microbiologic identification. Our findings demonstrate that *Rickettsia* species first encountered in tick surveys are associated with human disease, and we should not assume that some *Rickettsia* species not have a pathogenic potential.
